# Digitally Delivered Dietary Interventions for Patients with Eating Disorders Undergoing Family-Based Treatment: Protocol for a Randomized Feasibility Trial

**DOI:** 10.2196/41837

**Published:** 2023-01-26

**Authors:** Megan Hellner, Dori Steinberg, Jessica H Baker, Camilla Blanton

**Affiliations:** 1 Equip Health Carlsbad, CA United States

**Keywords:** eating disorders, virtual treatment, dietary interventions, family-based treatment, anorexia, psychiatric disorder, digital health intervention, telehealth, virtual health

## Abstract

**Background:**

Eating disorders (EDs) affect 9% of the United States population, and anorexia nervosa (AN), specifically, has the second highest mortality rate of all psychiatric disorders. Yet, only 20% are able to access treatment. Access to care issues include long waitlists, lack of trained specialists, financial, and geographic barriers, all of which highlight the need for effective telehealth interventions. Family-based therapy (FBT) is a first-line treatment for adolescents and young adults with EDs, and weight gain early in treatment is considered a primary predictor of success with FBT. However, nutrition requirements for patients with EDs are uniquely complex. A variety of dietary interventions for guiding the renourishment process are used in practice, but empirical data on the effectiveness and acceptability of the various interventions are sparse. The significance of nutritional restoration and issues with access to first-line treatments underscore the need for further research exploring virtually delivered dietary interventions.

**Objective:**

Our objective is to compare the effectiveness and acceptability of 2 digitally delivered dietary interventions frequently used in eating disorder treatment settings: (1) calorie-based meal plans and (2) the Plate-by-Plate approach. Specifically, we will explore any potential differences in weight restoration achieved over 8 weeks of treatment as a primary measure of effectiveness, as well as additional treatment outcomes (ED symptoms, anxiety, depression, caregiver burden, and perceived effectiveness and acceptability for both caregivers and clinicians).

**Methods:**

Patients (N=100) with either AN or avoidant restrictive food intake disorders (ARFID) aged 6-24 years seeking treatment at a nationwide virtual eating disorder treatment program, were enrolled between May and August 2022. Upon admission, patients were randomly assigned to receive either the calorie-based intervention or Plate-by-Plate approach from their registered dietitian, all of whom have received training as study interventionists. While we were primarily interested in responses during the first 8 weeks of treatment, patients will be followed for up to 12 months. Descriptive statistics were used to describe patient characteristics and demographics. Weight changes and other treatment outcomes between groups will be compared using generalized linear models. Semistructured caregiver and clinician interview transcripts will undergo qualitative analysis.

**Results:**

Enrollment ran from March to August 2022, and we anticipate completion of data collection by November 2022. Analyses will be completed in January 2023.

**Conclusions:**

This study contributes to existing FBT literature by thoroughly exploring the acceptability of dietary interventions and their influence on weight restoration, an area in which research is sparse.

**International Registered Report Identifier (IRRID):**

DERR1-10.2196/41837

## Introduction

### Background

Eating disorders (EDs) are serious mental illnesses characterized by how one experiences a marked disturbance in weight and shape [1], afflicting people across all ages, genders, races, ethnicities, and body sizes. National prevalence estimates are 9%, and EDs have the second highest mortality rate (specifically anorexia nervosa [AN]) following opioid addiction [2]. The economic burden is also substantial, with an annual cost of US $64.7 billion [3]. Further, the COVID-19 pandemic has resulted in a staggering increase in new ED cases, as well as exacerbation of ED symptoms and decreased motivation for recovery for those with preexisting EDs [4,5]. The rise in demand and challenges with access to care were compounded by long waitlists, lack of trained specialists, financial and geographic barriers, and underscored need for improving access to effective ED treatments [6].

Telemedicine or digitally delivered ED treatment options offer an opportunity to meet the rising demand while addressing geographic barriers and providing patients with timely access to trained specialists and reduce barriers associated with accessing care. Telemedicine ED treatments have been on the rise primarily as a result of the COVID-19 pandemic, and recent research supports their effectiveness and comparability to in-person care [7-11]. Family-based treatment (FBT) is an approach through which caregivers are tasked with renourishing their child at home while given guidance by a team of specialists. FBT is a first-line treatment for adolescents and young adults with AN and shows promise as an effective modality for other ED subtypes as well [12,13]. Recent research supports the efficacy of digitally delivered FBT as outcomes rival those of in-person treatment options [14-16]. Nutritional restoration is a central treatment element across all ED subtypes, and weight gain in the first several weeks of FBT is considered a primary predictor of remission [17,18]. However, nutrition requirements for patients with EDs are uniquely complex and varied, with kilocalorie needs reaching 70-100 kcal per kg per day, and caregivers generally require ongoing support to successfully navigate home-based refeeding. The importance of nutritional restoration for remission highlights the need for a better understanding of the dietary interventions most commonly used in ED treatment settings. While the inclusion of the registered dietitian (RD) on multidisciplinary teams is standard in other ED treatment settings and modalities aside from FBT, historically, FBT was led by a family therapist who was also responsible for giving guidance around feeding. In recent years, the need for specialized nutrition support has become more apparent, and the presence of RD is more commonplace. The RD can skillfully address issues that arise in treatment (management of malnutrition, refeeding syndrome, weight plateaus, erroneous belief systems around food and weight, lack of diversity in food choices, and needs adjustments for patients return to sport) that may be unaddressed by therapists, but are vital for achieving remission [19,20]. Additionally, the RD assists by giving guidance on optimization of energy density, determination of goal weight range, alleviation or management of physical symptoms that arise secondary to refeeding, and identification of nutrients of concern, which is of particular importance for patients with avoidant restrictive food intake disorder (ARFID) [21]. However, a variety of dietary interventions are used in practice across the ED field, and the evidence comparing effectiveness of dietary approaches, in person or digitally delivered, is sparse.

### Dietary Interventions

An overview of the interventions most commonly used in practice when treating ED patients are as follows: 1. Daily Calorie Target: caregivers or patients are provided a daily kilocalorie goal, and generally, the number of meals and snacks will also be prescribed, each with an associated kilocalorie goal. Provision of a daily calorie goal and discussions of calories in foods are generally discouraged in settings, where dietary interventions are patient-facing, as doing so may exacerbate ED thoughts and behaviors. Calorie targets may, however, be provided to caregivers as a means of guiding home-based refeeding efforts.

2. Plate-by-Plate approach: the Plate-by-Plate is a visual approach with primary intent for use in FBT, whereby caregivers are instructed to serve “normal” portions or plates versus using calories to guide meal or snack planning (Multimedia Appendix 1). This approach is regarded as a more flexible and intuitive, as calorie counting and measuring are discouraged. For patients requiring weight restoration, caregivers are instructed to create a plate consisting of 50% starch, 25% protein, and 25% vegetables and fruits, with dairy and fat servings specified by the RD for the meals. Key features of this approach include primary caregivers making the decision around what and how much to serve, with an emphasis on dietary variety and exposure to all foods. These features are thought to be important as they uphold parental empowerment, a core tenet of FBT [22].

3. Exchange system: this approach is most widely used in residential and partial hospitalization programs, where guidance is given directly to the patient [22]. The exchange system was developed by the American Diabetes Association as a way to categorize foods into 6 groups, and each “exchange” in a given food group is associated with a particular macronutrient range [23]. For example, 1 dairy exchange can be 1 oz of cheese or 8 oz milk. A number or range of exchanges are assigned to each meal and snack, thereby encouraging the patient to consider balance and dietary variety. The exchange system is seldom used in FBT given the different needs of caregivers versus patients.

This feasibility study will compare the acceptability of 2 digitally delivered dietary interventions and preliminary effectiveness in early stages of treatment: (1) calorie-based meal plans and (2) the Plate-by-Plate approach [24]. Our primary outcome of interest is weight restoration achieved after 8 weeks of treatment, as this is the best predictor of remission. We will also examine the following treatment outcomes by intervention: ED symptoms, anxiety, depression, caregiver burden and self-efficacy, and caregiver’s acceptability and perception of effectiveness. Acceptability and perceived effectiveness will also be measured for clinicians once week 8 outcomes have been captured for all participants. While we are most interested in early treatment response, patients will be followed and clinical outcomes for patients and caregivers reported throughout the entirety of their treatment course as part of standard practice. We anticipate that the findings from this study will help us better understand the needs and experiences of caregivers undergoing FBT and ultimately, whether 1 approach is superior in terms of influence on treatment outcomes. We will also gain insight around commonly held assumptions, such as the running hypothesis that providing caregivers with a calorie target may undermine parental empowerment and self-efficacy. This study will add to existing FBT literature by exploring the impact of dietary interventions that guide caregivers' process for renourishing their child, for which there is currently no research.

## Methods

### Participants

We will examine the aspects of treatment outcome in patients aged 6-24 years self-selecting for web-based ED treatment at Equip Health. The inclusion criteria are as follows: (1) aged 6-24 years, (2) ED diagnosis of AN or ARFID, (3) consented to participate in treatment approach comparison research upon admission, (4) apparent need for weight restoration upon admission, (5) first time recipients of treatment at Equip Health, and (6) assigned to RD interventionist.

### Setting

This single-site study takes place at Equip Health. Equip Health provides web-based ED treatment to children and adolescents ages 6-24 years across the United States using an FBT approach.

### Ethical Considerations

This study was reviewed by the Biomedical Research Alliance of New York (BRANY) institutional review board (protocol number 22-016-1096), and it was determined that it does not meet the criteria for human subject’s research as this is a quality improvement study of Equip Health treatment. Thus, institutional review board oversight was not required. Patients or caregivers consented to participate in treatment approach comparison studies upon admission and consented for treatment information to be evaluated and disseminated. Data will be stored in Equip’s Health Insurance Portability and Accountability Act–compliant medical record platform and will be deidentified upon extraction by trained research personnel. Interviews with the RD interventionists will be conducted via a Health Insurance Portability and Accountability Act–compliant Zoom platform. The final study results will be reported in aggregate, and will not contain any personally identifiable information.

### Recruitment

Patients seeking treatment at Equip inquire through Equip’s web-based form through direct email or phone. We randomized 100 eligible patients who enrolled in ED treatment on a rolling basis from May through August 2022.

### Group Assignment

As part of standard of care at Equip Health, all patients receiving FBT are assigned an RD as part of their multidisciplinary treatment team. Upon admission, patients eligible for this study and who consented to be a part of treatment comparison studies at Equip will be randomly assigned using a block design to receive either the calorie-based approach or the Plate-by-Plate approach during renourishment treatment with the RD. Participating patients were randomized for the purpose of having an equal number of participants in each treatment group. All other treatment procedures followed the standard of care for all Equip patients.

### Assessments and Measures

#### Overview

Outcome measures are described below and summarized in Table 1. Additionally, the following demographic data will be extracted from the medical record: age, state of residence, biological sex, gender identity, ED diagnosis, and height. Patients will be followed for up to 12 months, and clinical assessment measures are taken throughout treatment as part of standard practice. However, our primary time point of interest is week 8 of treatment, as this will provide important information about the patient’s response in the early phases of treatment, a critical time period for predicting remission.

**Table 1:** Primary and secondary outcome measures.

Measure		Outcome	Frequency assessed and timepoints of interest
**Primary** **outcome**			
	Weight	Weight change	Twice weekly (baseline, week 8)
**Secondary outcomes**			
	Eating Disorder Examination Questionnaire Short Form	Global total score, Eating concern scale score, Weight concern scale score, and Shape concern scale score	Upon admission, weekly thereafter (baseline and week 8)
	Burden Assessment Scale	Caregiver burnout	Every 40 days (baseline and week 8)
	Parent versus Eating Disorders	Caregiver self-efficacy	Day 14, every 40 days thereafter (baseline and week 8)
	Patient Health Questionnaire	Symptoms of depression	Upon admission, monthly thereafter (baseline and week 8)
	Generalized Anxiety Disorder Scale	Symptoms of anxiety	Upon admission, monthly thereafter (baseline and week 8)
	Clinician semistructured interview	Acceptability and perceived effectiveness of intervention	End of study
	Caregiver semistructured interview	Acceptability and perceived effectiveness of intervention	Treatment week 8 (approximate)

#### Weight

Upon admission, caregivers receive instructions from their assigned Equip medical provider on proper procedure for checking weight. Weights are taken in the morning, after voiding, prior to any oral intake and patients are asked to wear light clothing. The patient’s weight (in pounds) is taken twice weekly by caregivers and communicated via text message from caregivers to Equip’s Health Insurance Portability and Accountability Act–compliant medical record platform. For each patient, the RD calculates a target weight for weight restoration. Target weight is calculated using expected weight data from each patient’s CDC BMI-for-age Growth Charts. Our primary end point of interest is weight change during the first 8 weeks of treatment, which will include an average weekly rate of weight gain and total weight change from admission to treatment week 8.

#### Eating Disorder Examination Questionnaire Short Form

The Eating Disorder Examination Questionnaire Short Form is an abbreviated 12-item version of the 28-item Eating Disorder Examination Questionnaire survey. The Eating Disorder Examination Questionnaire Short Form is a well-validated and reliable measure, which assesses ED symptom severity. Scores range from 0 to 36, with higher scores suggesting increasing severity of ED behavior [25].

#### Patient Health Questionnaire

The Patient Health Questionnaire-9 is a brief, well-validated, and reliable diagnostic tool for depression. Patients are asked to rate 9 items pertaining to depression symptoms they have found bothersome over the last 2 weeks from “0” (not at all) to “3” (nearly every day). Higher scores indicate greater levels of depression [26].

#### Generalized Anxiety Disorder Scale

The Generalized Anxiety Disorder Scale is a widely used, validated, and reliable self-report tool for screening, diagnosing, and assessing the severity of an anxiety disorder. Patients are asked to rate 7 items pertaining to anxiety symptoms, which are scored from 0 to 3, and the score is totaled. Higher scores indicate increasing severity of anxiety [27].

#### Burden Assessment Scale

The Burden Assessment Scale is a 19-item validated questionnaire that focuses on specific objective and subjective caregiver consequences of individuals with mental illness. Ten items assess objective burden (eg, financial problems) and 9 items assess subjective aspects of burden (eg, feelings and attitudes). Caregivers were asked to indicate the extent to which they experienced the described problem on a 4-point scale, with higher scores indicating greater levels of caregiver burden [28,29].

#### Parent versus Eating Disorders

Parental self-efficacy will be assessed with the Parent versus Eating Disorders, which was adapted from the Parent Versus Anorexia scale. The Parent versus Eating Disorders was designed to capture caregivers’ belief in their ability to overcome their loved one’s eating disorder. Seven items are ranked on a 5-point scale with higher scores indicating greater self-efficacy [30].

#### Caregiver Semistructured Interviews

A semistructured interview (Multimedia Appendix 2) will be completed with caregivers to assess their experience with the assigned renourishment strategy. Caregivers will be contacted directly by a member of the research team via their preferred email address to schedule and the interviews will be conducted via phone. If the caregiver does not respond to contact efforts, a Health Insurance Portability and Accountability Act–compliant Google Forms link will be provided via preferred email through which the caregiver can answer the interview questions, after which efforts to reach the caregiver will be discontinued.

#### Clinician Semistructured Interviews

A semistructured interview (Multimedia Appendix 3) with the RD interventionist will also be completed to assess the interventionists’ general experience with each approach. Interviews will be completed by a member of the Equip research team and completed remotely via Health Insurance Portability and Accountability Act–compliant Zoom platform.

### Statistical Analysis

The treatment outcomes will be collected via our Health Insurance Portability and Accountability Act–compliant medical record platform and will be extracted by trained research personnel. Descriptive statistics will be used to describe patient characteristics and demographics of our patients and treatment outcomes defined in Table 1. To examine any differences in effectiveness between the 2 interventions, we will compare the rate of weight change between the groups using generalized linear models in R version 4.1.1 (R Core Team). We will also look at outcomes across ED subtypes.

### Procedures

Upon beginning treatment at Equip Health, eligible patients will be randomly assigned to receive either the calorie-based approach or the Plate-by-Plate intervention within their first 1-2 sessions with their assigned RD. In the first phase of treatment, the RD meets exclusively with caregivers (eg, parents or guardians) at a weekly meeting cadence for the first month, and thereafter in accordance with the RD’s judgment.

A high-level summary of each approach is as follows: (1) calorie-based nutrition prescription: following the completion of a nutrition assessment by the RD, caregivers will be prescribed a calorie goal and be instructed to aim to reach that goal daily as a primary focus of treatment. In collaboration with caregivers, a calorie goal will be assigned to 3 meals and 3 snacks daily. For example, the goal may be 3000 kcal per day, comprised of 700 kcal meals 3 times per day, and 300-kcal snacks 3 times per day. Additionally, caregivers are provided 7-day sample menus that align with their calorie prescription. If clinically indicated, they may also receive education on how to increase the caloric density of meals while maintaining low volume. Adjustments will be made throughout the treatment course to calorie prescriptions per RD’s judgment to accommodate the weight restoration goal of 2 lbs per week. During the initial session, caregivers are instructed to send photos and density estimates of all foods consumed by the child to the RD, and feedback will be given speaking to the adequacy of calorie content of meals and snacks in the follow-up sessions. (2) Plate-by-Plate approach is summarized for caregivers in the following steps:

1. Choose 10-inch plate
2. Plate all food groups
3. Fill plate
4. Parent decides number of meals or snacks
5. Include variety
6. Does the meal make sense?
7. Final review: Does the plate make sense?

Caregivers are instructed to prompt the patient to complete the plate, and in the case of food refusal, oral supplements are provided to replace the remaining food.

### Training and Fidelity

All Equip RDs have experience in treating EDs using a variety of dietary interventions within and outside of the FBT framework. Upon hiring, Equip RDs complete an in-depth video training on various interventions commonly used in ED treatment, including the Plate-by-Plate approach and calorie-based meal plans. RD interventionists undergo a live training session overviewing study protocols and each intervention, and a prerecorded training on the Plate-by-Plate intervention (led by authors of the Plate-by-Plate intervention). Interventionists will receive ongoing training as needed and supervision to ensure adherence to the respective approaches and will additionally be trained to notify research staff of any fidelity issues or protocol deviations by other providers on a treatment team.

Electronic records will be reviewed by study personnel on a weekly basis to ensure adherence to the assigned treatment approach. Specifically, session documentation, chat communications, and any document exchanges will be reviewed. Any fidelity issues (and the subsequent potential need for participant withdrawal) will be discussed by research personnel and interventionists. Further, should a patient discharge prior to week 8 of treatment, or if the RD determines that the assigned intervention is a poor fit for the patient, the patient may be withdrawn.

## Results

### Demographics

A total of 100 patients (with average age of 15 years) were enrolled on a rolling basis from March through August 2022. Fifty patients were assigned to the Plate-by-Plate group and 50 to the calorie target group. Overall, 83% of participants were cisgender females, 9% were cisgender males, 6% were nonbinary, and 2% were transgender. Furthermore, 66% of participants were diagnosed with AN restricting subtype, 18% with the AN binge-purge subtype, and 16% with ARFID. We anticipate completion of data collection by November 2022 and analysis by January 2023.

### Significance

We anticipate that, following completion of this study, we will be able to (1) address common myths, assumptions, and misconceptions associated with each intervention; (2) identify differences in outcomes and emerging themes from interview data, and use findings to adapt interventions and the supporting digital resources; (3) identify a preferred, or “best practice” approach for improving outcomes in the early phase of FBT, as early response is predictive of remission; and (4) understand any potential differences or nuances that may exist by intervention across the 2 ED subtypes (AN and ARFID).

### Design Considerations

While a variety of interventions may be used in practice, we selected Plate-by-Plate and calorie-based approaches due to their common use in FBT. Discussions of calories and the use of calorie targets to guide renourishment are generally discouraged in ED treatment settings in an effort to decrease preoccupations with calorie counting. Building upon this hypothesis, Plate-by-Plate authors cite the following rationale for use of a visual approach: (1) informed by a study that found an association between calorie-tracking application use and either maintenance or worsening of ED symptoms [31], authors hypothesized that a visual and simplistic versus numbers-based approach may have a beneficial influence on eating disorder symptoms and would allow for a seamless transition to “normal,” independent eating in later stages of treatment and beyond. Further, any calorie counting done by caregivers may “trickle down” to the patient and may thereby reinforce or exacerbate ED symptoms or thoughts. (2) A visual approach offers more flexibility by accommodating dietary restrictions, culturally appropriate food preferences, and easy adaptation for meals and snacks consumed outside of the home. (3) Given the lack of nutritional guidance due to exclusion of RDs in early FBT models, Plate-by-Plate was created to help parents with refeeding efforts using a more specialized approach, while still honoring the “parental empowerment” tenant central to FBT by allowing parents to select foods served.

With this study, we aim to test and challenge these assumptions through direct comparisons with a calorie-based approach. As part of standard practices at Equip Health, digital “food journals” (ie, photos and descriptions of foods) are provided by caregivers for assessment by RDs. In the absence of photos, the calorie-based approach allows parents to share calorie totals, which we suspect offer a more precise estimate of intake, and are less subject to parent interpretations and verbal description of portions or foods served and consumed. A calorie-based target may offer flexibility in “how” needs are met, and, like Plate-by-Plate, honors cultural preferences and dietary restrictions. We will further test the assumption that calorie targets trickle down to the patient, as caregivers are explicitly instructed not to share calorie information with patients, and ED patients have an existing general awareness of calorie content of foods and are able to quickly estimate upon provision of a meal or snack. Further, it is common practice in FBT to encourage parents to prepare and plate foods without the child present to minimize negotiations, resistance, and opportunity for patients to scrutinize components or portions.

### Participant Selection

The decision was made to exclude ED subtypes other than AN and ARFID for the following reasons:

1. Need for weight restoration was an important part of the inclusion criteria as weight restoration is typically first priority and a core component of ED treatment. Given that we are less likely to encounter a need for weight restoration in other ED subtypes, and would thus yield a small sample size within those subtypes, we opted to include only patients with AN and those with ARFID, which are the primary presenting subtypes in our patient population. In addition, while caregiver feedback is important, various aspects of FBT can be challenging for caregivers. We felt it important to have an outcome measure (weight) that is most predictive of remission and success with FBT.
2. In addition to there being little research on dietary interventions in the ED space in general, research looking at dietary interventions for ARFID, and particularly for patients undergoing FBT, is especially sparse. Given the differences in symptoms and presentation between patients with ARFID and those with AN, we felt it is important to better understand the benefits and challenges associated with the use of a visual approach. For example, patients with ARFID may present with a very limited number of acceptable foods and the lack of variety may make it challenging for parents to present meals or snacks with multiple components from numerous food groups. It is our hope that the outcome data, coupled with rich insights from clinicians and caregivers, will help us understand which aspects of our interventions may be most appropriate for patients with ARFID, and the ways in which they differ from patients with AN.

## Discussion

### Strengths and Limitations

To our knowledge, this will be the first comparison of digitally delivered dietary interventions for the treatment of EDs. Given access issues faced by patients seeking ED treatment, and increasing acceptability and use of telehealth, it is of particular value to test interventions in the digital space. Testing assumptions upon which approaches are developed is also valuable as we were able to gain insights that help us tailor approaches that better resonate with caregivers and providers.

It should be noted that the majority of patients who present for ED treatment at Equip have engaged with numerous prior programs and providers, and as such, they may have encountered various dietary approaches. While it was not realistic nor warranted to exclude those with prior treatment histories, we addressed this by instructing RD interventionists to notify study personnel should it prohibitive to orient caregivers to the assigned approach. At that point, a determination will be made as to whether the patient should be withdrawn from the study. Further, although with FBT caregivers are tasked with renourishing their child between ages 6 and 24 years, it is possible that there may be differences in intervention across age ranges, particularly for patient-facing interventions. However, it should be noted that individual (patient) nutrition therapy is provided only in later stages of FBT (generally following several months of nutritional restoration).

### Future Work

In this study, we compare 2 digitally delivered dietary interventions most commonly used by dietitians in eating disorder treatment settings and subsequent outcomes. Future research is needed to examine the effectiveness of interventions delivered directly to patients and across the broad range of ED subtypes.

## Appendix

Multimedia Appendix 1 Semistructured registered dietitian interventionist interview script.

Multimedia Appendix 2 Semistructured caregiver interview script.

Multimedia Appendix 3 Overview of Plate-by-Plate approach.

## Abbreviations

AN: anorexia nervosa
ARFID: avoidant restrictive food intake disorder
ED: eating disorder
FBT: family-based treatment
RD: registered dietitian

## Data Availability

The data that will support the findings of this study will be derived from patient medical record data. The data are not publicly available and individual data cannot be shared due to privacy and institutional restrictions.
