# Supplementary material for: Digitally Delivered Dietary Interventions for Patients with Eating Disorders Undergoing Family-Based Treatment: Protocol for a Randomized Feasibility Trial
Source: JMIR Res Protoc. 2023 Jan 26;12:e41837. doi: 10.2196/41837 (PMC9912149; doi:10.2196/41837)
Supplement: Multimedia Appendix 1 [file resprot_v12i1e41837_app1.docx]

**Multimedia Appendix 1: Semi-Structured RD Interventionist Interview Questions**

Purpose: To assess ease of use, thoughts, attitudes and perceived effectiveness of each approach.

Introduction: We would like to ask you about your experiences implementing the Plate-by-Plate and Calorie Based renourishment approaches with your patients. I am going to ask you a few questions related to your viewpoint on the ease of use, effectiveness, and your general thoughts and attitudes of each approach.

1. How would you describe the pros and cons of the Plate-by-Plate approach as they pertain to you as a provider? Please include thoughts around how easy it was to teach to caregivers, how easy it was to make adjustments, caregiver receptivity and effectiveness.
2. How would you describe the pros and cons of the Calorie approach as they pertain to you as a provider? Please include thoughts around how easy it was to teach to caregivers, how easy it was to make adjustments, caregiver receptivity and effectiveness.
3. Describe your approach and experience giving guidance to the patient directly in phase II of FBT. How did that differ by approach used by family?
4. Thinking about the caregiver experience, How would you describe the pros and cons of Plate by Plate in thinking about the caregiver experience?
5. Thinking about the caregiver experience, how would you describe the pros and cons of the Calorie approach in thinking about the caregiver experience?
6. Did you notice any differences or similarities across ED subtypes?
7. Describe which approach felt more successful and why? Which do you feel was more effective, and why?
8. What surprised you most about using each approach with caregivers?
9. Do you have any other thoughts or insights you’d like to share? Please share any other insights or thoughts here.
10. On a scale from 1-10, with 1 being worst, 5 being average, and 10 being best how would you rank:

1. The effectiveness of the Plate by Plate approach for weight restoration?
2. The effectiveness of the Calorie based approach for weight restoration?
3. The ease of implementing the Plate by Plate approach in session?
4. The ease of implementing the Calorie based approach in session?
5. Caregiver receptivity to the Plate by Plate approach?
6. Caregiver receptivity when using the Calorie based approach?

1. All other things equal, did you prefer the Plate by Plate or Calorie based approach?
