# Supplementary material for: Digitally Delivered Dietary Interventions for Patients with Eating Disorders Undergoing Family-Based Treatment: Protocol for a Randomized Feasibility Trial
Source: JMIR Res Protoc. 2023 Jan 26;12:e41837. doi: 10.2196/41837 (PMC9912149; doi:10.2196/41837)
Supplement: Multimedia Appendix 2 [file resprot_v12i1e41837_app2.docx]

**Multimedia Appendix 2: Semi-Structured Caregiver Interview**

Introduction/Directions: Occasionally we ask our patients about their experiences in treatment so that we can make improvements in the treatment we provide. I’d like to ask you just a few brief questions about your sessions with your registered dietitian, specifically about the skills and techniques discussed around how to re-nourish your child.

1. Was your child using a Calorie based plan, or a visual (Plate-by-Plate) approach with your RD?
2. How would you describe your experience initially (at start of treatment) with the approach your RD used?
3. How did your thoughts on/feelings about the approach change over time from week 1 until now?
4. What do you think are the pros and cons of the approach?
5. Do you have suggestions for how we could have adapted or changed the approach to make it easier and more effective for you?
6. In your opinion, what kind of method or approach would be most effective for caregivers who are engaging in treatment for an eating disorder and whose child needs to restore weight?
7. On a scale of 1-5, with 1=’very ineffective’’ and 5=’very effective’, how effective do you feel this approach was in helping your child restore weight?
8. On a scale of 1-5, with 1=’very difficult to use’ and 5=’very easy to use’, how easy do you feel this approach was for you to learn and implement/use?

****In the event the interview questions are sent in survey form via HIPAA compliant Google Forms, the interview questions above will be entered as is and caregivers will be asked to provide responses via free text.**
