# Supplementary material for: Digitally Delivered Dietary Interventions for Patients with Eating Disorders Undergoing Family-Based Treatment: Protocol for a Randomized Feasibility Trial
Source: JMIR Res Protoc. 2023 Jan 26;12:e41837. doi: 10.2196/41837 (PMC9912149; doi:10.2196/41837)

THE PLATE-BY-PLATE APPROACH®

ESSENTIAL STEPS AND STRATEGIES

STEP 1: CHOOSE A 10”-SIZED PLATE

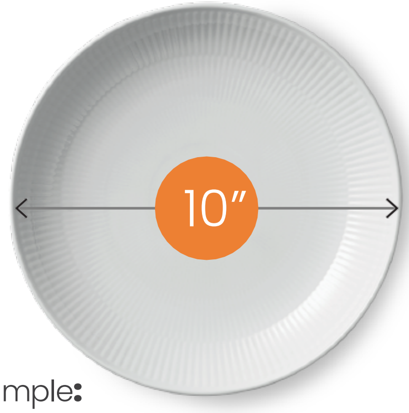

STEP 2: PLATE ALL FOOD GROUPS and IN THE RIGHT RATIO

Grain/Starch (33% plate) | **Protein** (33% plate) | **Fruit/Veggie** (33% plate)

Fat (added while cooking and visible), Dairy (Serving)

STEP 3: Plate FULL plates - 100% FULL. Eat every 2-4 hours – For example:

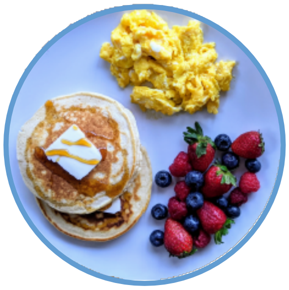

7:00 AM
Breakfast

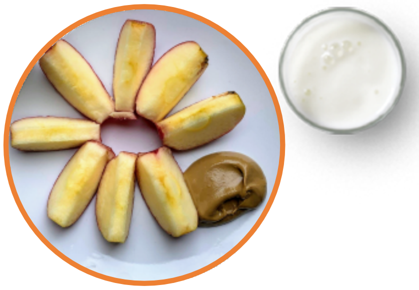

10:00 AM
Morning Snack

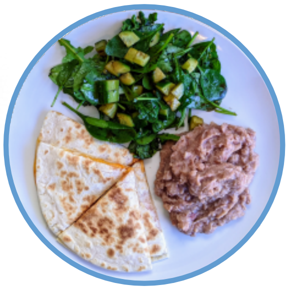

12:00 PM
Lunch

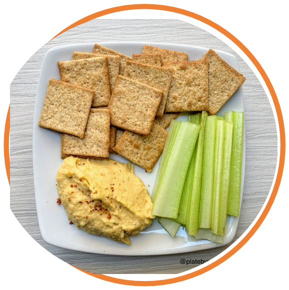

3:00 PM
Afternoon Snack

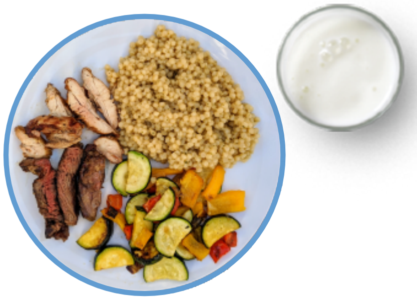

6:00 PM
Dinner

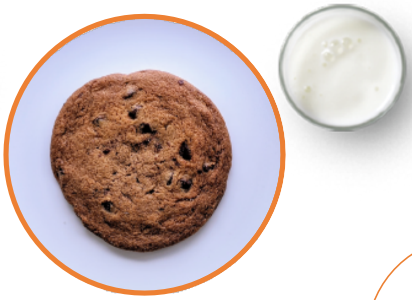

9:00 PM
Evening Snack

Snacks: At least 2 different food groups or 3 if one of the food items is a fruit or veg. Evenly spaced.

- ☐ Snacks should be served 2-3 times/day
- ☐ Recommended for all – with/without ED
- ☐ Helps regulate metabolism, blood sugars, provides fuel for sports (if cleared)

STEP 5: INCLUDE VARIETY:

Often your child's "preferences" and "eating disorder" can get tangled up.

Think back to what your child liked before the onset of their eating disorder and incorporate those foods.

Exposure is key!

STEP 6: DOES THE MEAL "MAKE SENSE?"

STEP 7: THE FINAL REVIEW: HOW DOES THE PLATE LOOK?

5 Final Check-Off Questions:

- ☐ Are all food groups present? Grains/Starches? Protein? Fruit/Vegetable? Dairy? Fat?
- ☐ Is the whole plate full?
- ☐ Have you challenged your child?
- ☐ Is the plate 33% grains/starches, 33% protein, 33% fruit/vegetable?
- ☐ Does the meal "make sense" and feel cohesive?

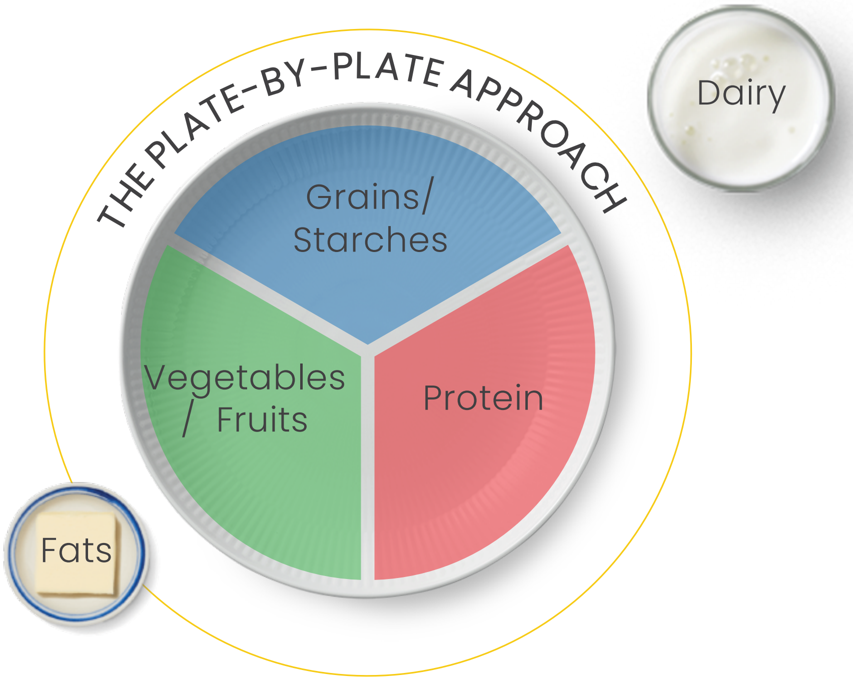

Supplement: Multimedia Appendix 3 [file resprot_v12i1e41837_app3.pdf]
